# Medical News and Miscellany

**Published:** 1888-02

**Authors:** 


### Medical News and Miscellany.

Dr. L. W. Groce, a graduate of the Galveston Medical College, is attending a course of lectures at the Jefferson Medical College, and will graduate there this spring. The Doctor is a warm friend of the Journal.

Dr. C. W. LeGrande, of Hempstead, Texas, is at the New York Polyclinc.

Dr. C. McReynolds, Fort Smith, Ark., has recently taken a special course in orthopædic surgery, and will contribute a series of articles to the Journal on that subject. His first paper will appear in the next issue, and will be on Talipes (*varus and vulgus*).

We are pleased to see that our friend Dr. Frank L. James, the able and well known microscopist and chemist, has assumed the editorial management of the *National Druggist*, of St. Louis, still maintaining his position as editor of the *St. Louis Medical and Surgical Journal*. The Doctor has his hands full, but he is equal to any emergency.

Dr. H. M. Whelpley, formerly editor of the *National Druggist*, has accepted the editorship of a new journal *The Druggist*, published at St. Louis by Meyer Bros.

THANKS.—We tender our thanks to Dr. F. E. Yoakum, President East Line Medical Association, at Greenville, for a club of six subscribers, with a check for the money. More are promised.

ASSESSMENT No. 5, P. M. B. A.—At the expiration of the thirty days (February 15,) one hundred members had paid the assessment, and one hundred dollars has been sent to Mrs. Starley. Second notices have been issued to those who have not paid, and the net result of the balance—estimated at about $30—will be forwarded when collected.

